# Pattern Recognition in Medical Decision Support

**DOI:** 10.1155/2019/6048748

**Published:** 2019-06-13

**Authors:** Shadnaz Asgari, Fabien Scalzo, Magdalena Kasprowicz

**Affiliations:** ^1^Biomedical Engineering Department, California State University, Long Beach, USA; ^2^Computer Engineering and Computer Science Department, California State University, Long Beach, USA; ^3^Department of Neurology, University of California, Los Angeles, USA; ^4^Department of Computer Science, University of California, Los Angeles, USA; ^5^Department of Biomedical Engineering, Wroclaw University of Science and Technology, Wroclaw, Poland

Recent advances in data acquisition and various monitoring modalities have resulted in generating and collecting a growing volume of biological and medical data at unprecedented speed and scale [1]. These accumulated data can be utilized for a more effective delivery of care and enhanced clinical decision-making [2]. However, analysis of these tremendous amounts of data—collected from electronic health records or monitoring devices—to extract useful information for a more broad-based health-care delivery is one of the main challenges of today's medicine [3].

Medical decision support systems help clinicians to best exploit these overwhelming amount of data by providing a computerized platform for integrating evidence-based knowledge and patient-specific information into an enhanced and cost-effective health care [4]. Over the last decade, various pattern recognition techniques have been applied to biomedical data (including signals and images) for automatic and machine-based clinical diagnostic and therapeutic support. The development of novel pattern recognition methods and algorithms with high performances, in terms of accuracy and/or time complexity, improves the health-care outcome by allowing clinicians to make a better-informed decision in a timelier manner. This is of vital importance especially when a rapid clinical decision needs to be made in a stressful environment such as intensive care units [5]. Development of predictive computational models and pattern recognition algorithms with performances and capabilities matching the complexity of rapidly evolving clinical measurement and monitoring systems is an ongoing research area and, thus, it requires continuous update on the current status of its advances [6].

With that scope in mind, this special issue focuses on the applications of pattern recognition for clinical decision support. For this purpose, we selected five research articles that discussed the design and clinical applications of feature extraction and/or classification of large-scale or high-dimensional biomedical data including biomedical signals and images.

S. Long et al. developed and evaluated an automatic retinal image processing algorithm to detect hard exudates (HE). Their algorithm consisted of four main stages: (i) image preprocessing; (ii) localization of optic disc; (iii) identification of potential HE region using dynamic threshold and fuzzy C-means clustering; and (iv) extraction of texture features from the HE region being fed into a support vector machine classifier. The proposed algorithm was trained and cross-validated on a publicly available e-ophtha EX database, achieving the overall average sensitivity of 76% and positive predictive value of 83%. Testing their algorithm on DIARETDB1 database resulted in better sensitivity (97%) and specificity (98%).

Z. Luo et al. developed and tested a machine learning approach to detect hypertension by automatic processing of radial artery blood pressure signals collected from healthy and hypertensive subjects by a noninvasive measurement device designed and built in house. They applied K-means clustering to exclude the noisy pulses from the data. Then they identified a subset of features that helped the most in detecting the hypertensive cases and applied various pattern classification algorithms such as AdaBoost and random forest. The authors were able to achieve the highest accuracy of 86% using AdaBoost.

K. Y. Win et al. proposed a novel methodology for the detection of cancer cells in cytological pleural effusion (CPE) images. Following some image intensity adjustment and application of median filtering to improve the image quality, cell nuclei were extracted by a hybrid segmentation method based on the fusion of simple linear iterative clustering and K-means clustering. Shape and contour concavity analyses were carried out to detect and split any overlapped nuclei into individual ones. Then several morphometric, colorimetric, and texture features were extracted. A novel hybrid feature selection method was developed using an artificial neural network to select the most discriminant and biologically interpretable features. Finally, an ensemble classifier of bagged decision trees was utilized to classify cells into being either benign or malignant. The proposed method achieved sensitivity of 88% and specificity of 99% on a dataset of 125 images containing more than 10,000 cells.

J. Jaworek-Korjakowska employed analysis of vascular structures in dermoscopy color images to distinguish benign and malignant pigmented skin lesions using a segmentation technique and convolutional neural network. The proposed method achieved sensitivity of 85% and specificity of 81%. The author concluded that small size and similarity of vascular structures to other local structures makes the segmentation and classification of dermoscopy color images challenging.

H. Tang et al. proposed employment of various features (from time/frequency domains) of phonocardiogram data and support vector machine for classifying normal and abnormal heart sound data. They applied correlation analysis to quantify discrimination level of the features and used support vector machine with radial basis kernel function for classification of phonocardiogram data from publicly available PhysioNet database. Their methodology achieved an average sensitivity of 88% and a specificity of 87%.

With upcoming technology upgrades in clinical measurement and monitoring systems, it is reasonable to assume that the cost of acquiring and storing biomedical data will decrease dramatically in the near future [1, 7]. This substantiates the need to further enhance the existing signal processing and pattern classification algorithms or develop novel ones to analyze the resulting complex data and help health-care providers in a variety of clinical decision-making processes [3]. The papers presented in this special issue outline some of the recently developed pattern recognition methodologies in clinical decision systems.
